# Risk Assessment of Bovine Major Histocompatibility Complex Class II *DRB3* Alleles for Perinatal Transmission of Bovine Leukemia Virus

**DOI:** 10.3390/pathogens10050502

**Published:** 2021-04-22

**Authors:** Liushiqi Borjigin, Chieh-Wen Lo, Lanlan Bai, Rania Hamada, Hirotaka Sato, Shuji Yoneyama, Anna Yasui, Sohei Yasuda, Risa Yamanaka, Munehito Mimura, Michihito Inokuma, Yasuo Shinozaki, Naoko Tanaka, Shin-Nosuke Takeshima, Yoko Aida

**Affiliations:** 1 Viral Infectious Diseases Unit, RIKEN, 2-1 Hirosawa, Wako, Saitama 351-0198, Japan; liushiqi.borjigin@vetmed.hokudai.ac.jp (L.B.); lanlan.bai@riken.jp (L.B.); hirosato@dokkyomed.ac.jp (H.S.); 2 Baton Zone Program, Nakamura Laboratory, RIKEN Cluster for Science, Technology and Innovation Hub, 2-1 Hirosawa, Wako, Saitama 351-0198, Japan; 3 Photonics Control Technology Team, RIKEN Center for Advanced Photonics, 2-1 Hirosawa, Wako, Saitama 351-0198, Japan; rogerwen80@gmail.com (C.-W.L.); rania_hamada@vet.svu.edu.eg (R.H.); 4 Laboratory of Global Infectious Diseases Control Science, Graduate School of Agricultural and Life Sciences, The University of Tokyo, 1-1-1 Yayoi, Bunkyo-ku, Tokyo 113-8657, Japan; 5 Department of Animal Medicine, Faculty of Veterinary Medicine, South Valley University, Qena 83523, Egypt; 6 Kenou Livestock Hygiene Service Center, Utsunomiya, Tochigi 321-0905, Japan; yoneyamas01@pref.tochigi.lg.jp; 7 Kumagaya Livestock Hygiene Service Center, Kumagaya, Saitama 360-0813, Japan; yasui.anna@pref.saitama.lg.jp (A.Y.); yasuda.sohei@pref.saitama.lg.jp (S.Y.); yamanaka.risa@pref.saitama.lg.jp (R.Y.); mimura.munehito@pref.saitama.lg.jp (M.M.); 8 Chuo Livestock Hygiene Service Center, Chiba 262-0011, Japan; m.inkm@pref.chiba.lg.jp; 9 Nanbu Livestock Hygiene Service Center, Kamogawa, Chiba 296-0033, Japan; y.shnzk14@pref.chiba.lg.jp (Y.S.); n.akbsh@pref.chiba.lg.jp (N.T.); 10 Department of Food and Nutrition, Jumonji University, Niiza, Saitama 352-8510, Japan; takesima@jumonji-u.ac.jp

**Keywords:** bovine leukemia virus, perinatal infection, dam, calf, *BoLA-DRB3* polymorphism, proviral load, disease suitability, disease resistance

## Abstract

Perinatal transmission plays a critical role in the spread of bovine leukemia virus (BLV) infection in cattle herds. In the Holstein breed, we previously identified BLV resistant and susceptible bovine leukocyte antigen (*BoLA*)-*DRB3* alleles, including *BoLA*-DRB3*009:02 and *014:01:01 with a low BLV proviral load (PVL), and *015:01 and *012:01 with a high PVL. Here, we evaluated the perinatal BLV transmission risk in dams with different *BoLA-DRB3* alleles. *BoLA-DRB3* alleles of 120 dam-calf pairs from five dairy farms in Japan were identified; their PVL was quantified using the BLV-Coordination of Common Motifs (CoCoMo)-qPCR-2 assay. Ninety-six dams were BLV-positive, and 29 gave birth to BLV-infected calves. Perinatal transmission frequency was 19% in dams with resistant alleles suppressed to a low PVL level, and 38% and 25% in dams with susceptible and neutral alleles that maintained high PVL levels, respectively. Notably, all calves with resistant alleles were BLV free, whereas 30% of calves with susceptible genes were infected. Thus, vertical transmission risk was extremely lower for dams and calves with resistant alleles compared to those with susceptible alleles. Our results can inform the development of effective BLV eradication programs under field conditions by providing necessary data to allow for optimal selection of dams for breeding.

## 1. Introduction

Bovine leukemia virus (BLV) belongs to the family Retroviridae (genus *Deltaretrovirus*) together with human T-leukemia virus types 1 and 2 (HTLV-1 and -2), and causes enzootic bovine leucosis (EBL), the most common neoplastic disease affecting cattle worldwide [1]. Approximately 70% of BLV-infected cattle are asymptomatic, a stage designated as aleukemic, whereas approximately 25%–30% and 1%–5% of BLV-infected cattle develop persistent lymphocytosis and B cell lymphoma, respectively, after several years of latency [1].

In 2012, 51 countries or territories regularly reported the presence of EBL infections to The World Organisation for Animal Health (OIE) [2]. Currently, after decades of systematic control and eradication approaches, most European countries and Oceania have eradicated BLV from their dairy herds [2,3]. However, in several countries where compulsory eradication or control strategies have not been implemented, the spread of BLV infection continues owing to the absence of effective treatments or vaccines. Recently, high BLV prevalence has been reported in the United States (US), China, Canada, Japan, etc. [4,5,6,7,8]. Thus, BLV infection commonly affects the cattle industry worldwide and causes considerable economic loss owing to premature death of animals by lymphomas [9], carcass condemnation at slaughter [10], reduction in milk yield [6,11,12], and decreased immunity [13], as well as effects on reproductive capacities [14] and longevity [6,12]. The economic loss due to reduced milk production of BLV-infected cattle alone was estimated at 525 million USD annually in the US dairy industry [11]. Additionally, it was estimated that the annual mean partial net revenue from BLV-infected dairy cattle was 635 CAD less than that from BLV-free cattle [15].

BLV is transmitted primarily through the transfer of infected lymphocytes and via horizontal and vertical routes [1]. Horizontal transmission of BLV occurs primarily by close contact with infected animals or via blood-sucking insects, such as tabanids and stable flies, [16] or via iatrogenic procedures, including the repeated use of individual needles, syringes, rectal palpation gloves, and dehorners [17,18]. Meanwhile, vertical transmission includes perinatal and postnatal infection. Vertical postnatal infection from cattle to calves occurs via colostrum and milk [19,20]; the infectious capacity of cells in milk from BLV-infected dams is currently estimated by ex vivo visualization of BLV infection [21]. Conversely, perinatal infection may occur in utero or in the birth canal [19,20]. Previously, Mekata et al. [19] investigated the frequency of perinatal BLV infection in field conditions in Japan and observed that 10 out of 129 (7.7%) calves born from BLV-infected cows were infected in the birth canal and 14 (10.8%) were infected *in utero*. Thus, perinatal transmission of BLV plays a critical role in the spread of BLV infection in cattle herds.

The BLV proviral load (PVL), which represents the amount of retroviral genome integrated into the host genome, strongly correlates with infection capacity, as assessed by syncytium formation [22,23], and with disease progression [5,22]. Cattle with high PVLs are considered major transmission sources [5,19,24,25] and risk factors for progression of EBL [5,22,26]. Additionally, studies on BLV-associated host factors have identified polymorphisms within the bovine major histocompatibility complex (MHC) (BoLA) [26,27,28]. BoLA is a highly polymorphic gene set that plays a central role in antigen recognition of pathogens and is, thus, used extensively as a marker of disease and immunological traits in cattle. Previously, we identified BoLA class II *DRB3* resistant alleles associated with low BLV PVL and susceptible alleles associated with a high PVL in Japanese black cattle and Holstein cattle [26,28,29]. However, whether *BoLA-DRB3* polymorphism is a risk factor for vertical postnatal infection of BLV remains unclear.

The previous association study demonstrated that, in Japanese Holstein cattle, *BoLA*-DRB3*002:01, *009:02, and *014:01:01 represent resistant alleles, while *BoLA*-DRB3*012:01 and *015:01 were identified as susceptible alleles for BLV PVL [28]. Therefore, in the current study we investigated the prevalence and PVL of BLV in dams and their calves to evaluate the perinatal BLV infection risk for cattle carrying these *BoLA-DRB3* alleles.

## 2. Results

### 2.1. Risk of Perinatal BLV Transmission in Dams with Different BoLA-DRB3 Genotypes

From January 2017 to March 2020, 120 calves were born at five dairy farms in Japan and immediately separated from their mothers and placed into individual calf hatches. Thereafter, they were given heat sterilized colostrum or commercial milk replacer to prevent horizontal and vertical postnatal BLV infection. We collected peripheral blood samples from the 120 dam-calve pairs (Table 1), and subsequently genotyped them to determine *BoLA-DRB3* allele polymorphisms (Table 2). We identified 14 of the previously reported *BoLA-DRB3* alleles (the Immuno Polymorphism Database (IPD)-MHC database (https://www.ebi.ac.uk/ipd/mhc/group/BoLA/ accessed on 15 April 2021)), of which two were resistant alleles, (*BoLA*-DRB3*009:02 and *014:01:01), and two were susceptible alleles (*BoLA*-DRB3*012:01 and *015:01). We then compared the frequency of *BoLA-DRB3* genotypes including resistance and susceptible alleles. Of the 120 dams, 17 (14.2%) had resistance and neutral allele genotypes, 5 (4.2%) had genotypes of resistance and susceptible allele genotypes, 6 (5.0%) had susceptible/susceptible allele genotypes, 49 (40.8%) susceptible/neutral allele genotypes, and 43 (35.8%) had neutral/neutral allele genotypes, respectively (Table 2 and Table 3). In particular, among the 22 dams with resistance alleles, 4 (3.3%), 13 (10.8%), and 5 (4.2%) had BoLA-DRB3*009:02/neutral alleles, BoLA-DRB3*014:01:01/neutral alleles, and BoLA-DRB3*014:01:01/susceptible alleles, respectively (Table 2). Further, among the 55 dams with susceptible alleles, 14 (11.7%), 35 (29.2%), 3 (2.5%), and 3 (2.5%) had BoLA-DRB3*012:01/neutral, BoLA-DRB3*015:01/neutral, BoLA-DRB3*012:01/015:01, and BoLA-DRB3*015:01/015:01, respectively (Table 2). Cumulatively, these results show that dams with susceptible and neutral alleles accounted for a large proportion.

**Figure 1:**
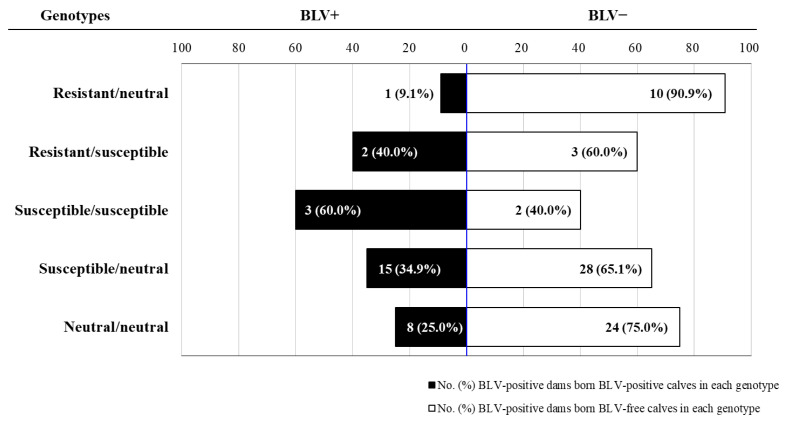
The number and ratio of dams with different bovine leukocyte antigen (*BoLA*)*-DRB3* genotypes. Black bars and white bars with black frames represent the numbers and ratios of dams that gave birth to bovine leukemia virus (BLV)-positive (BLV^+^) or BLV-negative (BLV^−^) calves in different genotypes.

Thereafter, to determine the BLV infection status of dams, we performed quantitative real-time polymerase chain reaction using Coordination of Common Motifs (CoCoMo-qPCR-2) to calculate the BLV PVL, and enzyme-linked immunosorbent assays (ELISAs) were used to estimate the anti-BLV antibody titer (Table 2). Ninety-six (80%) of the 120 dams were positive for BLV PVL and anti-BLV antibodies (Table 2 and Table 3). In contrast, we detected BLV PVL only using CoCoMo-qPCR-2 to determine BLV infection in calves. Twenty-nine (30%) of the 96 BLV-positive dams gave birth to calves that were positive for BLV PVL (Table 2 and Table 3). Notably, 62% of the 29 dams gave birth to BLV-positive calves that carried susceptible/susceptible genotypes (10.3%) and susceptible/neutral genotype (51.7%; Table 3). Thus, the population of dams that gave birth to BLV-positive calves carrying the susceptible alleles was higher than the population of total dams carrying the susceptible/susceptible (5.0%) and susceptible/neutral genotypes (40.8%) and the population of BLV-positive dams carrying the susceptible/susceptible (5.0%) and susceptible/neutral genotypes (49.0%). In contrast, the population of dams that gave birth to BLV-positive calves with resistant/neutral genotypes (3.4%) and resistant/susceptible genotypes (6.9%) was lower than the population of all dams (18.4% = 14.2% + 4.2%) and BLV-positive dams (16.7% = 11.5% + 5.2%) carrying the same genotypes. Notably, three dams gave birth to BLV-positive calves carrying the BoLA-DRB3*014:01:01 alleles (BoLA-DRB3*014:01:01/neutral and BoLA-DRB3*014:01:01/*015:01), but not to those carrying the BoLA-DRB3*009:02 alleles (Table 2). Figure 1 shows the frequency of BLV-positive dams or BLV-negative dams in five distinct genotypes. The proportion of BLV-positive dams with resistant/neutral genotypes (9.1%) was markedly lower than with the other four genotypes, whereas the frequency of BLV-positive dams with susceptible/susceptible genotypes (60%) were the highest compared to that of BLV-positive dams with the other four genotypes. These results show that dams with resistant alleles have a lower risk of vertical BLV transmission than those with susceptible alleles.

### 2.2. Distribution of PVLs in Dams with Different BoLA-DRB3 Genotypes

**Figure 2:**
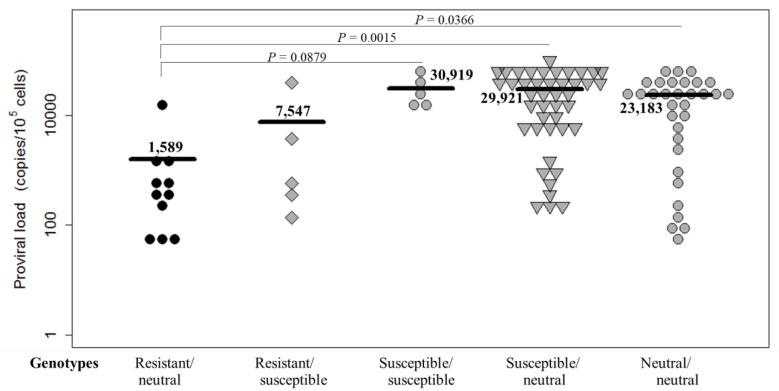
The bovine leukemia virus (BLV) proviral load (PVL) of 96 BLV-infected dams with different bovine leukocyte antigen (*BoLA*)*-DRB3* genotypes, sampled from five farms in Japan from January 2017 to March 2020. PVL in peripheral blood was quantified using CoCoMo-qPCR-2 and *BoLA-DRB3* alleles were identified with the PCR-sequence-based typing method. The mean PVL was compared among five groups. *p* < 0.05 represents statistically significant and 0.05 < *p* < 0.1 represents tends to be significant, respectively.

The maternal viral load has been shown to significantly correlate with the frequency of perinatal infection [19]. Therefore, we further analyzed the distribution of PVLs among dams carrying the five genotypes (Figure 2). A total of 96 dams were positive for BLV, with PVLs ranging from 43 to 83,036 copies per 10^5^ cells, as determined by CoCoMo-qPCR-2 (Table 2 and Figure 2). Notably, 11 dams carrying resistant/neutral genotypes had the lowest PVL, ranging from 43 to 12,544 copies per 10^5^ cells (mean 1589 copies), among the five genotype groups. In contrast, five dams carrying susceptible/susceptible genotypes showed the highest PVL, ranging from 12,157 to 52,072 copies per 10^5^ cells (mean 30,919 copies), among the five genotype groups. In addition, PVLs of 43 dams carrying susceptible/neutral genotypes ranged from 205 to 83,036 copies/10^5^ cells (mean 29,921 copies per 10^5^ cells) and those of 32 dams carrying neutral/neutral genotypes ranged from 66 to 78,079 copies/10^5^ cells (mean 23,138 copies per 10^5^ cells); these dams (*p* = 0.0015 for susceptible/neutral genotypes and *p* = 0.0366 neutral/neutral genotypes genotypes) had significantly higher PVLs than those carrying resistant/neutral genotypes. Thus, our results showed that the PVLs in dams carrying resistant alleles were lower than those in dams carrying susceptible and neutral alleles.

### 2.3. Frequencies of BLV Provirus in Calves with Different BoLA-DRB3 Genotypes

**Figure 3:**
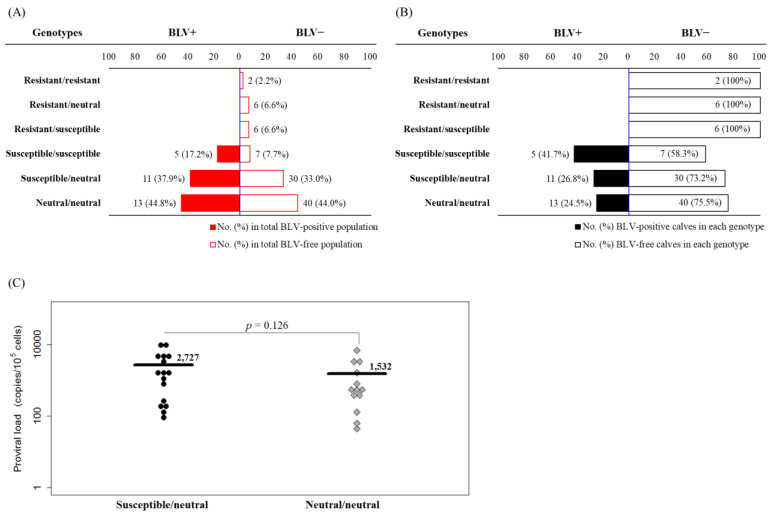
Estimation of bovine leukemia virus (BLV) vertical transmission probability by dams with different bovine leukocyte antigen (*BoLA*)*-DRB3* genotypes. (**A**) Red and white bars with red frames represent the number and ratio of calves with each *BoLA-DRB3* genotype between the total of BLV-positive calves (BLV^+^) and the total of BLV-negative calves (BLV^−^). (**B**) Black and white bars with black frames represent the number and ratio of the probabilities of vertical transmission of BLV by calves with different *BoLA-DRB3* genotypes. (**C**) PVL in peripheral blood from calves was quantified using CoCoMo-qPCR-2, and *BoLA-DRB3* genotypes were identified with the PCR-sequence-based typing method. The mean PVL was compared between two groups with different *BoLA-DRB3* genotypes and significant differences between both groups were calculated using Tukey’s test. *p* > 0.05 represents statistically not significant.

A total of 120 newborn calves were assessed for BLV infection and BLV PVL using CoCoMo-qPCR-2. Twenty-nine out of 96 BLV-positive dams gave birth to calves that were positive for BLV PVL (Table 3). In contrast, all 24 BLV-negative dams delivered calves that were negative for BLV PVL (Table 2), suggesting successful avoidance of postnatal vertical infection. Furthermore, genotyping was performed to determine the polymorphisms of BoLA-DRB3 alleles in these calves resulting in the identification of 14 of the previously reported alleles of the BoLA-DRB3 locus (Table 4). Among 29 BLV-positive calves, 5 (17.2%) had susceptible/susceptible genotypes, 11 (37.9%) had susceptible/neutral genotypes, and 13 (44.8%) had neutral/neutral genotypes (Figure 3A). Notably, all calves with three genotypes including resistant alleles were BLV free (Figure 3A). Conversely, among the 91 BLV-negative calves, 2 calves (2.2%), 6 calves (6.6%), 6 calves (6.6%), 7 calves (7.7%), 30 calves (33.0%), and 40 calves (44.0%) had resistant/resistant, resistant/neutral, resistant/susceptible, susceptible/susceptible, susceptible/neutral, and neutral/neutral genotypes, respectively (Figure 3A). Similarly, Figure 3B shows the frequency of BLV-positive or BLV-negative calves in the five distinct genotypes. Notably, no BLV-positive calves contained one of the three genotypes including resistant alleles. In contrast, the frequencies of BLV-positive calves with susceptible/susceptible, susceptible/neutral, or neutral/neutral genotypes ranged from 24.5% to 41.7%. Although PVL levels did not differ significantly between the 16 calves with susceptible alleles and 13 calves with only neutral alleles (*p* = 0.126), it tended to be higher in calves with susceptible/neutral genotype than in those with neutral/neutral genotype (Figure 3C). These results show that cattle with susceptible alleles are at a high risk for vertical transmission due to their genetic preference for maintaining a high level of PVL.

### 2.4. Differential Risk of BLV Vertical Transmission in Dams and Calves with Different BoLA-DRB3 Genotypes

**Figure 4:**
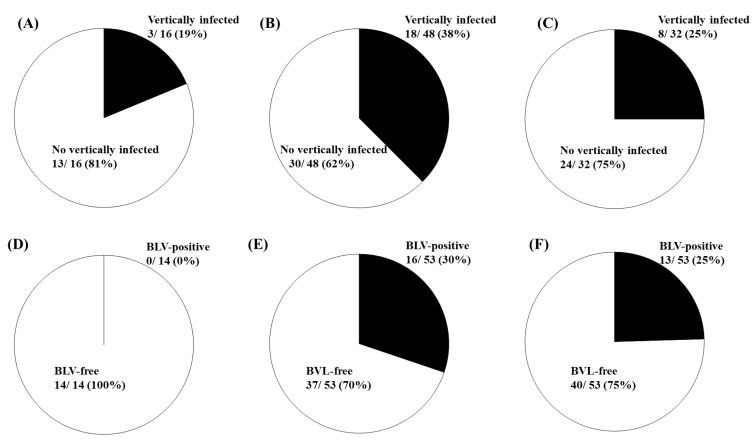
Probability of vertical bovine leukemia virus (BLV) transmission by dams and calves with different bovine leukocyte antigen (*BoLA*)*-DRB3* alleles. Black colored areas represent the probability of vertical transmission of BLV by BLV-positive dams (**A**–**C**) and calves (**D**–**F**) with different genotypes. PVL in peripheral blood from dams and their calves was quantified using CoCoMo-qPCR-2, and *BoLA-DRB3* alleles were identified by the PCR-sequence-based typing method. The probability of vertical transmission of BLV from BLV-positive dams with resistant/neutral and resistant/susceptibility genotypes, possessing at least one resistant allele (**A**); with susceptible/susceptible and susceptible/neutral genotypes, possessing only susceptible allele or susceptible and neutral alleles (**B**); and with neutral/neutral genotypes, possessing only neutral alleles (**C**). The probability of vertical transmission of BLV in BLV-positive calves with resistant/resistant, resistant/neutral, and resistant/susceptibility genotypes, possessing at least one resistant allele (**D**); with susceptible/susceptible and susceptible/neutral genotypes, possessing only susceptible allele or susceptible and neutral alleles (**E**); and with neutral/neutral genotypes, possessing only neutral alleles (**F**).

We compared the effect of BoLA-DRB3 alleles on BLV vertical transmission in dams and calves. As summarized in Figure 4A–C, the risk of BLV vertical transmission was low in dams with two genotypes (resistant/susceptible and resistant/neutral) possessing at least one resistant allele (19%), moderate in dams with genotype (neutral/neutral) possessing only one neutral allele (25%), and high in dams with two genotypes (susceptible/neutral and susceptible/susceptible) possessing susceptible and neutral or only susceptible alleles (38%). Similarly, the risk of vertical BLV transmission from infected dams to their calves was: 0% for calves with three genotypes (resistant/resistant, resistant/susceptible and resistant/neutral) possessing at least one resistant allele; 25%, for calves with neutral/neutral genotype possessing only a neutral allele; and 30% for calves with susceptible/neutral and susceptible/susceptible genotypes possessing susceptible and neutral or only susceptible alleles (Figure 4D–F).

## 3. Discussion

This is the first study to demonstrate that cattle with different *BoLA-DRB3* alleles have different risks of vertical transmission of BLV. In particular, we found that the risk of vertical transmission for dams and calves with resistant alleles was much lower than that for dams and calves with susceptible alleles. In addition, dams with susceptible alleles were at a higher risk for vertical transmission because of their genetic characteristics that maintain PVL at a high level, whereas PVL was maintained at a low level in most dams with resistant alleles, thereby reducing the risk of vertical BLV transmission.

Here, we successfully quantified the PVL of 120 dam-calve pairs from five dairy farms from January 2017 to March 2020 and showed the distribution of PVLs in dams and calves with different *BoLA-DRB3* genotypes. PVL was maintained at a low level in most dams with resistant alleles, except for one dam with DRB3*014:01:01/*015:01 (33,529 copies per 10^5^ cells) that consistently had a PVL above 10,000 copies per 10^5^ cells. Meanwhile, the two dams with DRB3*009:02 maintained a PVL lower than 200 copies per 10^5^ cells. These results show that DRB3*009:02 is the strongest resistant allele for BLV, while DRB3*014:01:01 is marginally weaker than DRB3*009:02. Previously, it was reported that BLV-infected cattle carrying the DRB3*009:02 allele is not a source of infection for BLV-free cattle [24]. Similarly, we demonstrated that cattle with resistant alleles do no undergo perinatal BLV transmission. This was first evidenced by the observation that 3.4% and 6.9% of the dams delivered BLV-positive calves with resistant/neutral and resistant/susceptible genotypes, respectively, which was significantly lower than the frequencies of dams that delivered BLV-positive calves carrying susceptible/susceptible (10.3%), susceptible/neutral (51.7%), and neutral/neutral (27.6%) genotypes. Second, only 9.1% of dams with resistant/neutral genotypes gave birth to BLV-positive calves; this was markedly lower than the proportion birthed by dams with the other four genotypes. Third, all calves delivered from BLV-infected dams with a genotype including resistant alleles, were BLV free. Conversely, dams with susceptible alleles had high PVLs, representing a major perinatal BLV infectious factor. In addition, frequencies among BLV-positive calves with three distinct genotypes, including susceptible/susceptible, susceptible/neutral, or neutral/neutral, alleles ranged from 24.5% to 41.7%, which were higher than that of calves with genotypes that include resistant alleles. PVL in calves carrying susceptible/neutral alleles tended to be higher than that of calves with neutral/neutral alleles. Previously, we reported that BLV proviruses were detected in the nasal secretions, saliva samples, and milk of individuals with high PVL in their blood [21,25,30]. Thus, our results demonstrate that cattle with susceptible alleles represent the most critical infectious agents in both horizontal and perinatal BLV transmission.

To investigate perinatal BLV transmission, we successfully tested the BLV for 120 dams and their calves within one month of delivery at five dairy farms from January 2017 to March 2020. To avoid postnatal vertical infection, all newborn calves were immediately separated from their dams and placed into individual calf hatches, and subsequently fed heat sterilized colostrum or commercial milk replacer during the study period. All 24 BLV-negative dams delivered calves that were negative for BLV PVL, indicating that postnatal infection can be avoided by implementing the above-mentioned countermeasures. In addition, the time from BLV infection to seroconversion is reported to be approximately 1–2 months [31]. Hence, we also performed testing for BLV infection within 1 month after delivery, as this is the optimal time to confirm perinatal infection.

Of the total 120 dams, the provirus was detected in 96 (80%). No provirus was detected in the 24 calves delivered from BLV free dams, indicating that no horizontal or postnatal vertical infections occurred prior to sampling. The perinatal infection rate in BLV-infected dams was confirmed to be 30%, which was higher than previously reported (18.6% in one farm in Japan) [19]. This may differ from maternal PVL or genetic characteristics at different farms or regions. The correlation between maternal PVL and the frequency of perinatal infection was previously reported [19]. We set a PVL of 10,000 copies/10^5^ cells as the cutoff for classification as high or low [26,28]. In this study, with the exception of two cases, it was clearly shown that dams with PVL > 6000 copies per 10^5^ cells were more susceptible to perinatal transmission to their calves. Twenty-seven of the 64 (42%) dams with PVL > 6000 per 10^5^ cells, while only two of the 32 (6%) dams with PVL < 6000 per 10^5^ cells, demonstrated perinatal infection. This agrees with data from previous studies that demonstrated > 40% of newborn calves were born to dams with high BLV PVL [19]. Although no correlation was found between PVL in dams and calves (data not shown), PVL in newborn calves with susceptible alleles tended to be higher than that in calves with neutral alleles. Thus, calves infected in the first week of life could play an active role in early propagation of BLV to susceptible cattle, as their PVL is increased during the first 12 months and is maintained for years [30]. Notably, all calves with resistant alleles were BLV free. These results suggest that the genetic characteristics of calves are involved in the increases in PVL after their birth and that culling them earlier is useful for effective BLV eradication.

As a result of identifying the distribution of *BoLA*-*DRB3* alleles in all 120 dams, 17 (14.2%), five (4.2%), six (5.0%), 49 (40.8%), and 43 (35.8%) dams had resistant/neutral, resistant/susceptible, susceptible/susceptible, susceptible/neutral, neutral/neutral, and genotypes, respectively. These results showed that the dams with susceptible alleles were the most prevalent. Conversely, the 96 BLV-infected dams comprised 29 dams that delivered BLV-positive calves and 67 dams that delivered BLV-negative calves. The proportion of dams with resistant/neutral genotypes was 17/120 (14.2%); 10/67 (14.9%) dams delivered BLV-negative calves and 1/29 (3.4%) dams delivered BLV-positive calves. Furthermore, the proportion of dams with susceptible/susceptible and susceptible/neutral genotypes was 55/120 (45.8%) dams; 30 (44.8%) dams delivered BLV-negative calves and 18 (62.1%) dams delivered BLV-positive calves. Moreover, the proportion of dams with neutral/neutral genotypes were 43 (35.8%) of all 120 dams, 24 (35.8%) dams delivered BLV-negative calves, and eight (27.6%) delivered BLV-positive calves. Notably, although the proportion of dams with susceptible/susceptible, susceptible/neutral, and neutral/neutral genotypes are similar among all 120 dams and dams that delivered BLV-negative calves, the proportion of dams with resistant alleles and susceptible alleles that delivered BLV-positive calves were lower and higher than that in all 120 dams and those that delivered BLV-negative calves, respectively. These results indicate that dams with resistant alleles have a lower risk of perinatal BLV transmission than dams with susceptible alleles. In addition, the frequency of perinatal BLV transmission was low (9%, 1/11) in dams with resistant alleles, moderate (25%, 8/32) in dams with neutral alleles, and high (38%, 18/48) in dams with susceptible alleles. This frequency in dams with resistant alleles was lower than that in dams with susceptible alleles.

The “test and segregate” or “test and cull” approaches have been considered most effective for BLV infection control or eradication [2,19]. However, the “test and cull” approach is applicable in farms with low or moderate transmission risk within a herd [2,15,32,33]. The “test and segregate” approach is inappropriate for farms with a smaller area where it is difficult to separate animals, and it may inconvenience management, milking work, etc. Furthermore, this approach makes it impossible to prevent vertical perinatal infection. Our findings clearly indicate that BLV-infected cattle with susceptible alleles represent the primary factor in vertical and horizontal transmission within herds. Therefore, selective breeding of cattle with BLV resistant *BoLA-DRB3* alleles, as well as the preferential culling of cattle with susceptible *BoLA-DRB3* alleles could reduce the risk of both horizontal and vertical transmission, and is useful for the development of an economically feasible and effective BLV eradication program under field conditions.

## 4. Materials and Methods

### 4.1. Clinical Animals

From January 2017 to March 2020, we collected peripheral blood from 120 dams and their calves (<1 month in age) at five dairy farms (A, B, C, D, and E) in Chiba, Saitama and Tochigi prefectures in Japan (Table 1). The A, B, C, D, and E farms had approximately 93, 70, 53, 70, and 126 Holstein cattle, respectively. The 120 newborn calves were immediately separated from their mothers and placed into individual calf hatches and subsequently fed heat sterilized colostrum or commercial milk replacer to prevent horizontal and vertical postnatal BLV infection.

### 4.2. Ethics Approval

This study was approved by the Animal Ethical Committee and the Animal Care and Use Committee of RIKEN (approval numbers H29-2-104 and W2019-1-001, respectively).

### 4.3. Collection of Blood Samples, Extraction of Genomic DNA, and Separation of Serum or Plasma

Genomic DNA was extracted from ethylenediaminetetraacetic acid (EDTA)-treated peripheral blood samples using the Wizard Genomic DNA Purification Kit (Promega corporation, Madison, WI, USA), according to the manufacturer’s instructions. The quantity and quality of extracted DNA was measured based on the A260/280 ratio using a Nanodrop One Spectrophotometer (Thermo Fisher Scientific, Waltham, MA, USA). Serum or plasma were separated from whole blood or EDTA-treated blood samples, respectively.

### 4.4. Enzyme-Linked Immunosorbent Assay (ELISA) for Anti-Env gp51 Antibody

BLV-specific Env gp51 antibodies were measured from serum or plasma samples using a BLV-specific antibody detection ELISA kit (JNC, Tokyo, Japan), according to the manufacturer’s instructions.

### 4.5. Quantification of BLV PVL Using the BLV-CoCoMo-qPCR-2 Assay

BLV PVLs were quantified using BLV-CoCoMo-qPCR-2 (RIKEN Genesis, Kanagawa, Japan) with THUNDERBIRD Probe qPCR Mix (Toyobo, Tokyo, Japan), as described previously [22,34]. In brief, a 183 bp sequence of the BLV LTR gene was amplified using the degenerate primer set “CoCoMo-FRW and CoCoMo-REV” and detected with a 15 bp 6-carboxyfluorescein (FAM)-labeled LTR probe. As the internal control, the *BoLA-DRA* gene was amplified using the primer set “DRA-F and DRA-R”, and detected with the FAM-labeled DRA probe. Finally, the PVL was calculated using the following formula: (number of BLV LTR copies/number of *BoLA-DRA* copies) × 10^5^ cells.

### 4.6. BoLA-DRB3 Genotyping

*BoLA-DRB3* alleles were typed using the PCR-sequencing-based typing (SBT) method [35]. Briefly, we amplified exon 2 of *BoLA-DRB3* with PCR using primers DRB3FRW and DRB3REV. Thereafter, the first PCR fragments were purified using an ExoSAP-IT PCR Product Purification Kit (USB Corp., Cleveland, OH, USA) and sequenced with the ABIPRISM BigDye Terminator Cycle Sequencing Ready Reaction Kit (Applied Biosystems, Foster City, CA, USA). Finally, sequence data were analyzed using Assign 400ATF ver. 1.0.2.41 software (Conexio Genomics, Fremantle, Australia).

### 4.7. Statistical Analysis

The Student’s *t*-test was used to determine the significance between the PVL of calves with susceptible and resistant alleles. Following analysis of variance, Tukey’s test was used to determine the significance in the PVL of dams with different alleles. The pairwise-prop-test was used to determine the significance in frequencies of perinatal BLV transmission from dams with different alleles, and in BLV prevalence of calves with different alleles. *p <* 0.05 was considered significant.

## 5. Conclusions

We have demonstrated that the risk of vertical transmission for dams and calves with resistant alleles was much lower than that for dams and calves with susceptible alleles. In addition, dams with susceptible alleles were found to maintain a high level of PVL, whereas PVL was maintained at a low level in most dams with resistant alleles. These results may contribute to the development of low-cost and high-efficiency BLV eradication strategies to reduce the BLV prevalence and the PVL via decreased selection of dams with susceptible alleles and high PVL, and increased selection of dams with resistant alleles and low PVL for breeding.

## Figures and Tables

**Table 1:** Number of dam-calf pairs from five farms that were sampled peripheral blood temporarily from January 2017 to March 2020.

	Sampling Year				
Farm	2017	2018	2019	2020	Total
A	2	5	2	0	9
B	2	2	0	0	4
C	2	3	0	0	5
D	5	3	11	0	19
E	16	18	32	17	83
Total	27	31	45	17	120

**Table 2:** BoLA-DRB3 alleles and proviral load (PVL) in 120 dams sampled from January 2017 to March 2020.

Dams Alleles	gp51 ^a^	PVL ^b^	Dams Alleles	gp51	PVL	Dams Alleles	gp51	PVL
**Resistant/Neutral Genotypes**						**Neutral/Neutral Genotypes**		
**009:02**/001:01	+^d^	189	**012:01**/011:01	+	17,625	011:01/007:01	+	78,079
**009:02**/001:01	+	47	**012:01**/011:01	+	11,459	011:01/011:01	+	**70,859**
**009:02**/010:01	−^e^	0	**012:01**/010:01	+	862	011:01/011:01	+	**57,379**
**009:02**/010:01	−	0	**012:01**/016:01	+	205	002:01/027:03	+	47,903
**014:01:01**/001:01	+	12,544	**012:01**/011:01	−	0	011:01/001:01	+	43,822
**014:01:01**/027:03	+	**^c^** ** 1491**	**015:01**/010:01	+	**83,036**	011:01/010:01	+	**42,597**
**014:01:01**/001:01	+	1181	**015:01**/011:01	+	72,853	011:01/007:01	+	**40,583**
**014:01:01**/027:03	+	674	**015:01**/001:01	+	67,185	010:01/001:01	+	35,257
**014:01:01**/011:01	+	490	**015:01**/007:01	+	65,839	010:01/010:01	+	31,068
**014:01:01**/007:01	+	428	**015:01**/011:01	+	**63,536**	011:01/027:03	+	28,112
**014:01:01**/011:01	+	321	**015:01**/001:01	+	54,091	011:01/010:01	+	26,038
**014:01:01**/011:01	+	68	**015:01**/011:01	+	**43,868**	011:01/011:01	+	25,977
**014:01:01**/011:01	+	43	**015:01**/011:01	+	42,759	010:01/010:01	+	25,839
**014:01:01**/027:03	−	0	**015:01**/027:03	+	41,824	011:01/010:01	+	25,547
**014:01:01**/007:01	−	0	**015:01**/001:01	+	**40,714**	011:01/010:01	+	25,547
**014:01:01**/011:01	−	0	**015:01**/011:01	+	38,416	001:01/001:01	+	**25,031**
**014:01:01**/011:01	−	0	**015:01**/011:01	+	38,371	010:01/001:01	+	22,180
**Resistant/susceptible genotypes**			**015:01**/011:01	+	33,300	010:01/010:01	+	20,134
**014:01:01/015:01**	+	**33,529**	**015:01**/001:01	+	24,249	011:01/027:03	+	18,607
**014:01:01/015:01**	+	3122	**015:01**/011:01	+	**22,690**	010:01/010:01	+	**13,613**
**014:01:01/015:01**	+	**360**	**015:01**/010:01	+	**20,238**	010:01/027:03	+	**11,914**
**014:01:01/012:01**	+	570	**015:01**/011:01	+	**16,800**	011:01/027:03	+	**10,928**
**014:01:01/012:01**	+	153	**015:01**/001:01	+	13,247	001:01/010:01	+	6876
**Susceptible/susceptible genotypes**			**015:01**/011:01	+	**9196**	010:01/001:01	+	3955
**012:01/015:01**	+	**52,072**	**015:01**/011:01	+	**7288**	010:01/001:01	+	1893
**012:01/015:01**	+	**28,311**	**015:01**/010:01	+	7063	010:01/001:01	+	794
**012:01/015:01**	−	0	015:01/010:01	+	**6252**	010:01/010:01	+	684
**015:01/015:01**	+	45,321	**015:01**/010:01	+	**6077**	011:01/001:01	+	223
**015:01/015:01**	+	**16,735**	**015:01**/011:01	+	6000	011:01/011:01	+	144
**015:01/015:01**	+	12,157	**015:01**/001:01	+	1727	011:01/001:01	+	109
**Susceptible/neutral genotypes**			**015:01**/001:01	+	1043	011:01/001:01	+	106
**012:01**/001:01	+	61,538	**015:01**/007:01	+	616	011:01/001:01	+	66
**012:01**/007:04	+	59,974	**015:01**/007:01	+	420	010:01/007:01	−	0
**012:01**/007:01	+	59,441	**015:01**/001:01	+	279	011:01/010:01	−	0
**012:01**/007:04	+	**57,550**	**015:01**/007:01	+	275	011:01/010:01	−	0
**012:01**/011:01	+	51,915	**015:01**/001:01	−	0	011:01/018:01	−	0
**012:01**/001:01	+	**44,098**	**015:01**/011:01	−	0	001:01/027:03	−	0
**012:01**/001:01	+	**42,750**	**015:01**/001:01	−	0	001:01/027:03	−	0
**012:01**/027:03	+	30,863	**015:01**/011:01	−	0	002:01/016:01	−	0
**012:01**/027:03	+	**19,058**	**015:01**/001:01	−	0	001:01/027:01	−	0
						011:01/007:01	−	0
						011:01/001:01	−	0
						001:01/007:01	−	0

^a^ Bovine leukemia virus (BLV)-positive cows were diagnosed using anant^a^Anti-Env gp51 antibodies was detected using the BLV antibody enzyme-linked immunosorbent assay (ELISA) Kit (JNC, Tokyo, Japan). ^b^ PVL was calculated using the BLV-Coordination of Common Motifs (CoCoMo)-qPCR-2 system (RIKEN Genesis, Kanagawa, Japan). PVL given as proviral copies per 10^5^ cells. ^c^ PVL of BLV-positive dams that delivered BLV-positive calves are given in boldface and underline. ^d^+, Positive. ^e^−, Negative.

**Table 3:** The proportions of dams with different genotypes, delivered BLV-positive calves and BLV-negative calves.

*BoLA-DRB3* Genotype	Dam no./Total no. (%)		Dam no. (%) with	
	All Dams ^a^	BLV-Positive Dams ^b^	BLV-Negative Calves ^c^	BLV-Positive Calves ^d^
Resistant/neutral	17/120 (14.2)	11/96 (11.5)	10/67 (14.9)	1/29 (3.4)
Resistant/susceptible	5/120 (4.2)	5/96 (5.2)	3/67 (4.5)	2/29 (6.9)
Susceptible/susceptible	6/120 (5.0)	5/96 (5.2)	2/67 (3.0)	3/29 (10.3)
Susceptible/neutral	49/120 (40.8)	43/96 (44.8)	28/67 (41.8)	15/29 (51.7)
Neutral/neutral	43/120 (35.8)	32/96 (33.3)	24/67 (35.8)	8/29 (27.6)
**Total**	120	96	67	29

“^a^” represent all dams sampled in this study. “^b^” represent the BLV-positive dams in this study. “^c^” represent the BLV-positive dams that delivered BLV-negative calves. “^d^” represent the BLV-positive dams that delivered BLV-positive calves.

**Table 4:** BoLA-DRB3 alleles and proviral load (PVL) in 120 calves, which were given birth from January 2017 to March 2020.

Calves Alleles	PVL	Calves Alleles	PVL	Calves Alleles	PVL
**Resistant/neutral genotypes**					
**009:02**/010:01	0	**015:01**/011:01	0	001:01/011:01	0
**009:02**/010:01	0	**015:01**/011:01	0	001:01/011:01	0
**009:02**/002:01	0	**015:01**/011:01	0	001:01/011:01	0
**014:01:01**/011:01	0	**015:01**/011:01	0	001:01/010:01	3721
**014:01:01**/011:01	0	**015:01**/010:01	3654	001:01/010:01	3255
**014:01:01**/001:01	0	**015:01**/010:01	0	001:01/010:01	374
**Resistant/resistant genotypes**		**015:01**/010:01	0	001:01/010:01	0
**014:01:01/014:01:01**	0	**015:01**/010:01	0	001:01/010:01	0
**014:01:01/014:01:01**	0	**015:01**/010:01	0	001:01/010:01	0
**Resistant/susceptible genotypes**		**015:01**/010:01	0	001:01/010:01	0
**014:01:01/015:01**	0	**015:01**/001:01	174	001:01/010:01	0
**014:01:01/015:01**	0	**015:01**/001:01	0	001:01/010:01	0
**014:01:01/015:01**	0	**015:01**/001:01	0	001:01/007:01	0
**014:01:01/015:01**	0	**015:01**/001:01	0	001:01/007:01	0
**014:01:01/015:01**	0	**015:01**/001:01	0	001:01/002:01	0
**014:01:01/012:01**	0	**015:01**/001:01	0	001:01/001:01	59
**Susceptible/susceptible genotypes**		**015:01**/001:01	0	001:01/001:01	0
**012:01/015:01**	4044	**015:01**/001:01	0	001:01/001:01	0
**012:01/015:01**	1496	**015:01**/007:01	0	001:01/001:01	0
**012:01/015:01**	0	**015:01**/002:01	0	001:01/001:01	0
**012:01/015:01**	0	**012:01**/011:01	0	001:01/001:01	0
**012:01/015:01**	0	**012:01**/011:01	0	001:01/001:01	0
**012:01/015:01**	0	**012:01**/010:01	1366	001:01/001:01	0
**015:01/015:01**	9988	**012:01**/010:01	246	001:01/001:01	0
**015:01/015:01**	662	**012:01**/007:01	4525	002:01/011:01	0
**015:01/015:01**	183	**012:01**/007:01	0	002:01/007:01	42
**015:01/015:01**	0	**012:01**/005:03	0	005:03/010:01	0
**015:01/015:01**	0	**012:01**/018:01	0	007:01/027:03	0
**015:01/015:01**	0	**012:01**/016:01	0	007:01/011:01	147
**Susceptible/neutral genotypes**		**Neutral/neutral genotypes**		007:01/007:01	0
**015:01**/027:03	5086	010:01/007:04	0	010:01/027:03	472
**015:01**/027:03	1096	011:01/027:03	8046	010:01/027:03	0
**015:01**/027:03	106	011:01/027:03	0	010:01/027:03	0
**015:01**/027:03	0	001:01/027:03	553	010:01/011:01	721
**015:01**/027:03	0	001:01/027:03	0	010:01/011:01	0
**015:01**/018:01	0	001:01/027:03	0	010:01/011:01	0
**015:01**/016:01	9355	001:01/027:03	0	010:01/010:01	0
**015:01**/016:01	0	001:01/027:03	0	011:01/010:01	0
**015:01**/011:01	1545	001:01/016:01	0	011:01/011:01	420
**015:01**/011:01	109	001:01/011:01	1562	011:01/011:01	0
**015:01**/011:01	0	001:01/011:01	545	011:01/011:01	0
**015:01**/011:01	0	001:01/011:01	0		

## Data Availability

The data presented in this study are available on request from the corresponding author.
